# Design and application of carbon nanomaterials for photoactive and charge transport layers in organic solar cells

**DOI:** 10.1186/s40580-016-0068-8

**Published:** 2016-04-15

**Authors:** Sunghwan Jin, Gwang Hoon Jun, Seokwoo Jeon, Soon Hyung Hong

**Affiliations:** 1 Department of Materials Science and Engineering, Graphene Research Center, KI for the Nano Century, Korea Advanced Institute of Science and Technology (KAIST), Daejeon, 305-701 Republic of Korea; 2 Center for Multidimensional Carbon Materials (CMCM), Institute of Basic Science (IBS), Ulsan, 689-798 Republic of Korea

**Keywords:** Organic solar cell, Carbon nanotube, Graphene, Photoactive layer, Charge transport layer

## Abstract

Commercialization of organic solar cell (OSC) has faltered due to their low power conversion efficiency (PCE) compared to inorganic solar cell. Low electrical conductivity, low charge mobility, and short-range light absorption of most organic materials limit the PCE of OSCs. Carbon nanomaterials, especially carbon nanotubes (CNTs) and graphenes, are of great interest for use in OSC applications due to their high electrical conductivity, mobility, and unique optical properties for enhancing the performance of OSCs. In this review, recent progress toward the integration of carbon nanomaterials into OSCs is described. The role of carbon nanomaterials and strategies for their integration into various layers of OSCs, including the photoactive layer and charge transport layer, are discussed. Based on these, we also discuss the prospects of carbon nanomaterials for specific OSC layers to maximize the PCE.

## Introduction

Over the past decade, organic solar cells (OSCs) have received a great deal of attention for their potential as an alternative clean energy source. The greatest advantage of OSCs over inorganic solar cells is their compatibility with low-cost, simple, solution processes for mass production [1–3]. Furthermore, the inherent light weight and flexibility of OSCs enables the production of portable energy devices. Despite these advantages, commercialization of OSCs has faltered due to their low power conversion efficiency (PCE) compared to conventional inorganic solar cells [4]. Low electrical conductivity, low charge mobility, and short-range light absorption of most organic materials limit the PCE of OSCs.

Conventional OSCs typically comprise two key layers (excluding the electrodes): a photoactive layer and a charge transport layer. To maximize the PCE in OSCs, the photoactive layer must effectively generate excitons with high optical absorption, and has good charge mobility for the carrier transport to both electrodes without recombination. High electrical conductivity with exclusive charge selectivity and proper bandgap alignment in the charge transport layer are critical to increase the survival rate of separated carriers.

Integration of carbon nanomaterials, such as carbon nanotubes (CNTs) and graphenes, is a promising way to overcome the limitations of conventional organic materials. CNTs and graphenes exhibit greater mobility and conductivity [5–11] than conducting polymers and can, thus, provide highly conductive pathways for carrier transport. The interface between carbon nanomaterials and polymers can generate spatial separation of the charges from the photoexcited carriers in the photoactive layer [12, 13]. Furthermore, semiconducting single-walled CNTs (SWNTs) exhibit a small energy bandgap and a broad absorption band that is expanded into the near-infrared range [14, 15]. As charge transport materials, optically transparent and highly conductive CNTs and graphenes can act as effective charge collection materials without preventing photon absorption in the photoactive layer. For these reasons, CNTs and graphenes would likely be excellent candidates for enhancing the performance of OSCs.

In this review, we present recent progress toward the integration of carbon nanomaterials in OSCs. Integration strategies and the role of carbon nanomaterials in each of layer of OSCs are described. Technological developments in the design of CNTs and graphenes for specific OSC layers to maximize the PCE are discussed. Based on these, we also discuss the prospects of carbon nanomaterials for OSCs.

## Review

OSCs contain a photoactive layer and charge transport layer excluding the electrodes, and the integration of CNTs and graphenes into these layers has been evaluated to enhance OSC performance. In this section, the key factors evaluated in recent studies to improve the design and integration strategies of CNTs and graphenes are reviewed.

### Integration of carbon nanomaterials in OSC photoactive layers

Integration of CNTs or graphenes in OSC photoactive layers typically involves the dispersion of CNTs or graphenes in electron-donating polymers. In these OSC designs, CNTs and graphenes act as electron acceptors and replace the conventional electron acceptors such as 6,6-phenyl-C_61_-butyric acid methyl ester (PCBM) or 6,6-phenyl-C_71_-butyric acid methyl ester (PC_71_BM). In OSCs, photons are absorbed in the photoactive layer and generate excitons (bound electron–hole pairs). These excitons diffuse into the photoactive layer with an exciton diffusion length of 5–20 nm [16], and dissociation of excitons occurs at the donor/acceptor interface. Then, separated holes and electrons are transported to the charge transport layer or electrodes via the electron donor or acceptor phase, respectively [17–20]. CNTs and graphenes can also provide exciton dissociation sites and charge pathways in the photoactive layer, and the higher conductivities and mobilities of CNTs and graphenes are greater than those characteristic of conventional electron acceptors.

**Fig. 1:**
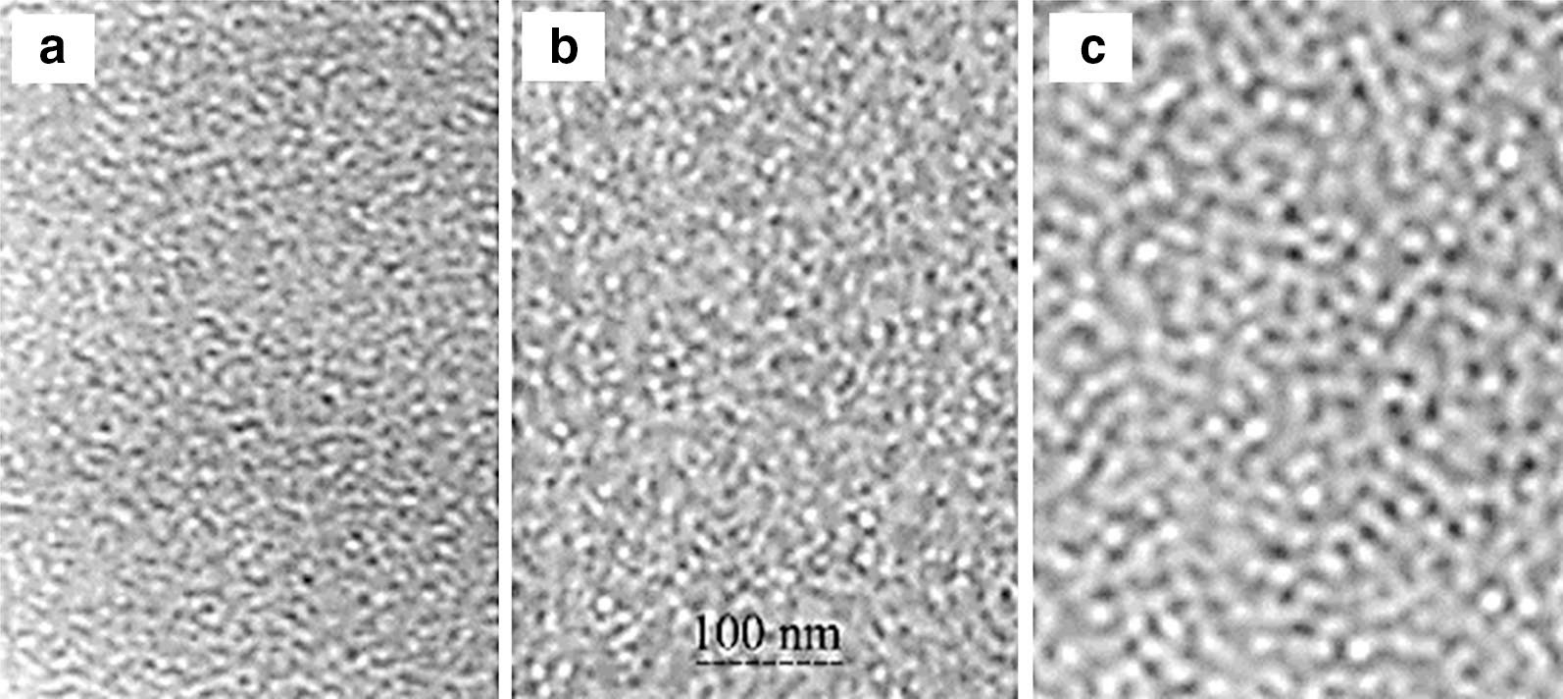
Transmission electron microscopy (TEM) images of poly-3-hexylthiophene (P3HT):6,6-phenyl-C61-butyric acid methyl ester (PCBM) **a** before thermal annealing, **b** after annealing at 150 °C for 30 min, and **c** after annealing at 150 °C for 2 h. Bright features correspond to PCBM, and dark features correspond to P3HT. PCBM and P3HT are clearly separated within 10 nm after thermal annealing. Reprinted with permission from Ma et al. [29], © 2005, Wiley

Many groups have reported the use of dispersed CNTs or graphenes as electron acceptors in electron-donating polymers like poly-3-hexylthiophene (P3HT) and poly-3-octylthiophene (P3OT) [21–28]. The highest PCEs reported for OSCs are 0.22 and 1.1 % using CNT/P3OT [24] and RGO/P3HT [25, 29–33] systems, respectively, as photoactive layers. However, these PCE values are lower than those reported for conventional P3HT/PCBM photoactive layer systems, which typically vary between 3 and 5 % [29–35]. Several factors may limit the performance of CNTs and graphenes as ele layers. First, a critical factor in bulk heterojunction (BHJ) photoactive layer systems is an ideal nanophase separation between donor and acceptor within the exciton diffusion length [36, 37], and technologies for such nanophase separation are lacking for CNTs and graphene photoactive layer materials. Various nanophase separation technologies for P3HT:PCBM systems have been well developed, including thermal annealing [29, 31, 34, 35, 38, 39] and solvent annealing [39, 40] as shown in the transmission electron microscopy (TEM) images in Fig. 1. However, CNTs and graphenes are randomly dispersed in electron-donating polymers. Moreover, the diameter of CNTs and the thickness of graphenes are nanoscale, but the length of CNTs and graphenes often reach the microscale. Agglomeration of CNTs and graphenes at high fractions can cause devices to short circuit easily, particularly considering that the total thickness of the OSCs is often less than 500 nm [21]. Second, CNTs and graphenes exhibit low carrier selectivity. The metallic properties of CNTs [41, 42] and graphenes [14, 42], which exhibit no bandgap for charge selection in OSCs, can lead to undesired charge recombination of holes and electrons during charge transport.

**Fig. 2:**
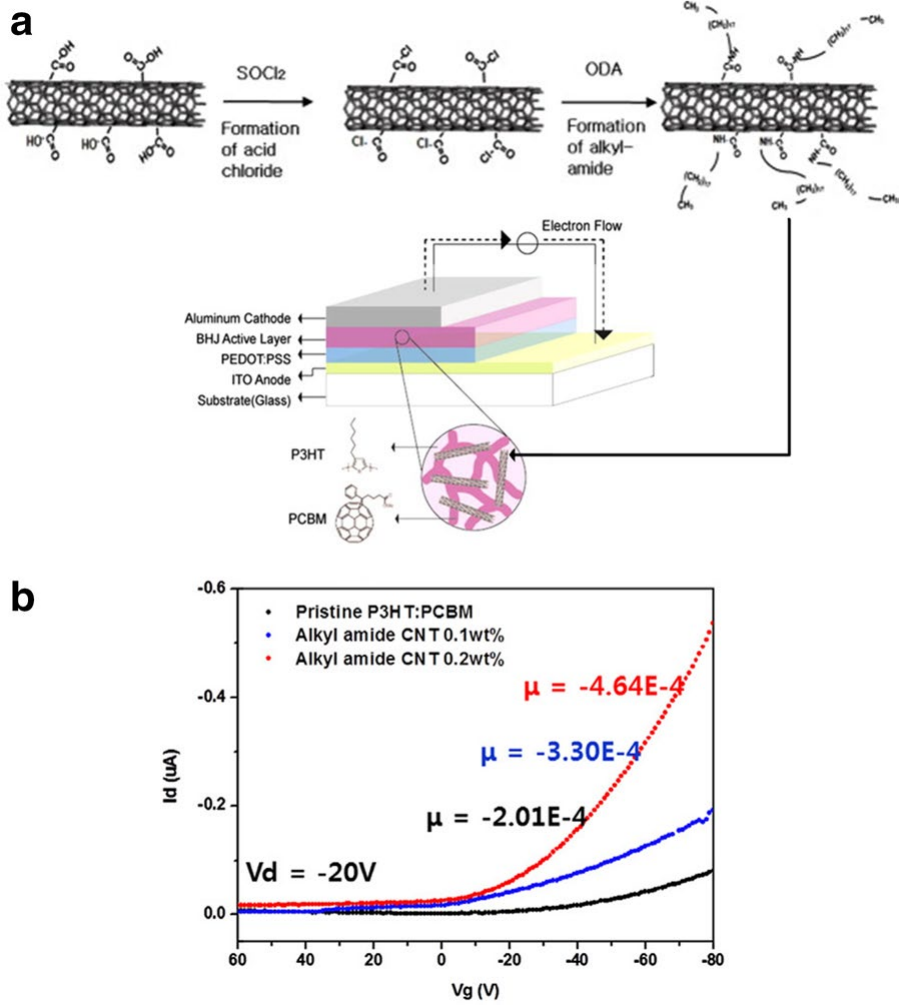
**a** A schematic representation of a carbon nanotube (CNT)/poly-3-hexylthiophene (P3HT):6,6-phenyl-C_61_-butyric acid methyl ester (PCBM) bulk heterojunction (BHJ) organic solar cell (OSC) and **b** the transfer characteristics of an organic thin film transistor made using a CNT/P3HT:PCBM composite. Alkyl amide functional groups are introduced to CNTs for a homogeneous dispersion in a matrix. μ = field-effect mobility of the organic thin-film resistor (OTFT; unit: cm^2^/Vs). Reprinted with permission from Jun et al. [44], © 2012, Elsevier

**Fig. 3:**
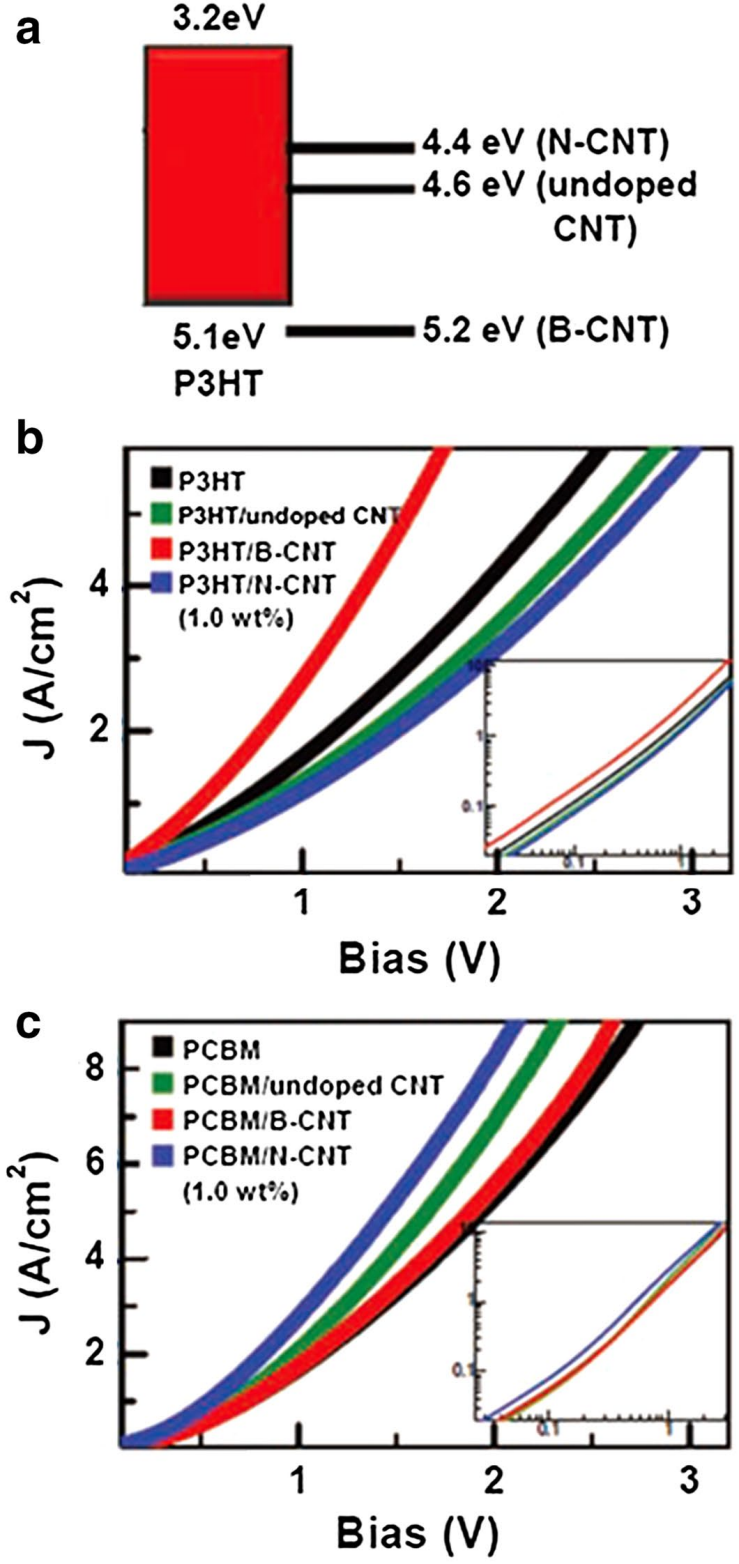
**a** A schematic energy diagram of poly-3-hexylthiophene (P3HT)/carbon nanotube (CNT) films, I-V characteristics of **b** electron-only and **c** hole-only devices. The slope of the I–V curve in logarithmic scale is shown in the two insets of (**b**) and (**c**). Reprinted with permission from Lee et al. [46], © 2011, Wiley

**Fig. 4:**
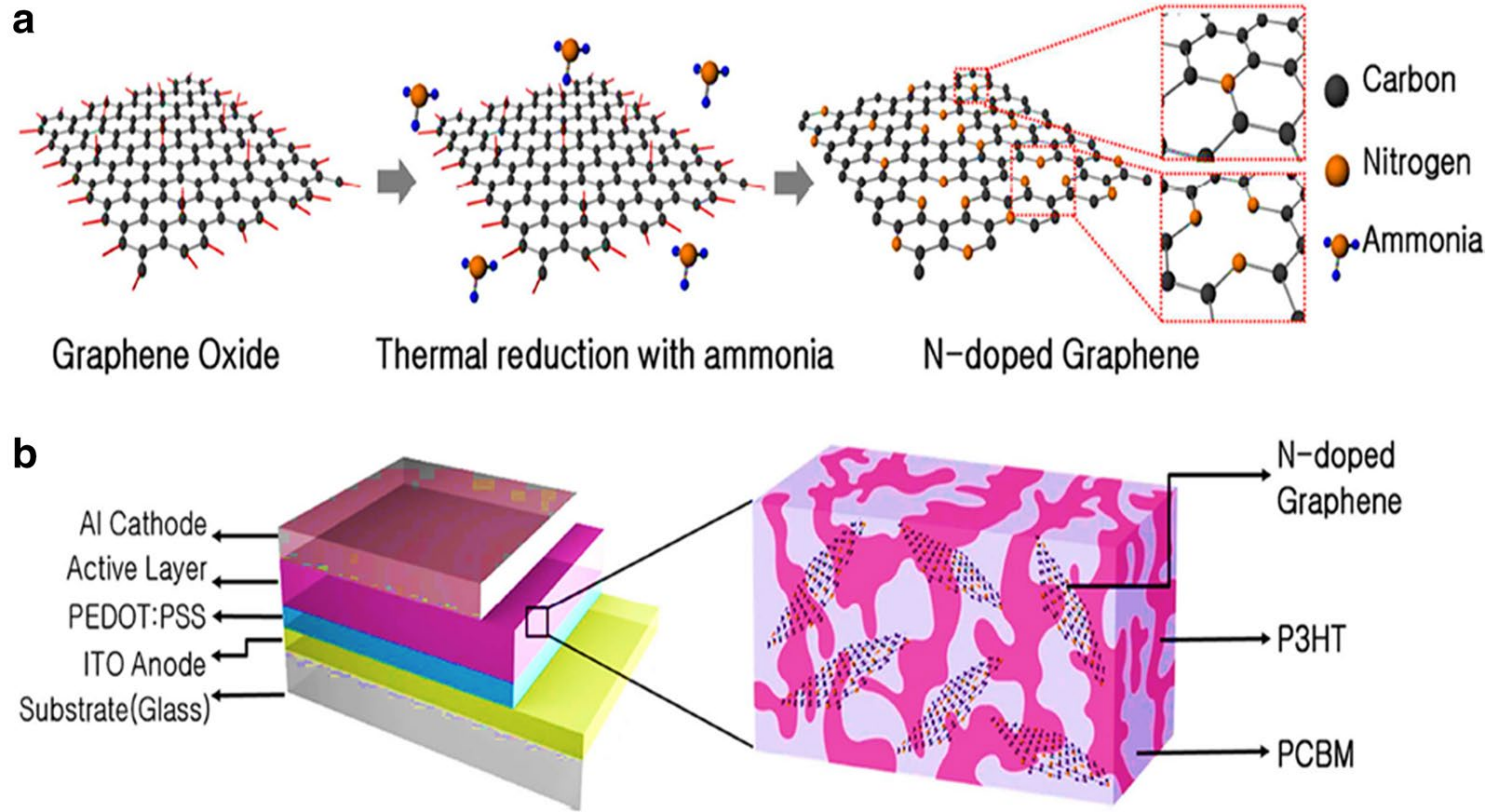
**a** A schematic depiction of the nitrogen doping process of reduced graphene oxide (RGO) and **b** a bulk heterojunction (BHJ) organic solar cell (OSC) made using a N-doped graphene/poly-3-hexylthiophene (P3HT):6,6-phenyl-C_61_-butyric acid methyl ester (PCBM) photoactive layer. Reprinted with permission from Jun et al. [47], © 2013, RSC

Several alternative strategies have been proposed to avoid these problems, such as the small addition of CNTs into a BHJ system [14, 43–45]. The addition of CNTs below 1 wt% in a P3HT/PCBM BHJ system did not negatively affect nanophase separation and increased the PCE up to 4.4 % [44] due to enhancement of carrier mobility, as shown Fig. 2. Furthermore, the addition of doped CNTs [46] and graphenes [47] to the BHJ system has also been suggested. Work function modulation of carbon nanomaterials upon doping can generate charge selectivity of carbon nanomaterials in OSCs. For example, Lee et al. [46] reported work function modulation of CNTs by nitrogen and boron doping. As shown in Fig. 3a, due to their respective energy levels in P3HT:PCBM systems, nitrogen-doped CNTs (N-CNTs) transport electrons, and boron-doped CNTs (B-CNTs) transport holes. Due to selectively enhanced carrier transport (Fig. 3b, c), the PCEs of OSCs upon the addition of 1 wt% doped CNTs are 4.1 and 3.7 % for B-CNTs and N-CNTs, respectively. These values are higher than those of OSCs with the addition of undoped CNTs (2.6 %). Furthermore, the highest PCE of about 8.6 % was achieved by adding of N-CNTs in the poly[[4,8-bis[(2-ethylhexyl)oxy] benzo[1,2-b:4,5-b′] dithiophene-2, 6-diyl] [3-fluoro-2-[(2-ethylhexy)carbonyl]thieno[3,4-b]thiophenediyl]] (PTB7): PC_71_BM BHJ system [48]. Jun et al. [47]. also reported the use of nitrogen-doped RGO (N-RGO) as an electron-selective transport material for P3HT:PCBM systems, as shown in Fig. 4. The PCE of an OSC using N-RGO exhibited a 40 % enhancement, from 3.2 to 4.5 %, compared to an OSC without N-RGO.

In contrast to the approach of CNTs as an electron acceptor in the photoactive layer, Hersam’s group reported CNTs as an electron donor in the CNT/PC_71_BM blend system [49]. They showed remarkable PCE over 3 % by using a mixture of n-type PC_71_BM and p-type semiconducting SWNTs, which are sorted from the synthesizing SWNTs by density gradient ultracentrifugation. It is a significant jump for the utilization of CNTs in the photoactive layer but still requires more studies or optimization to achieve higher PCE.

**Fig. 5:**
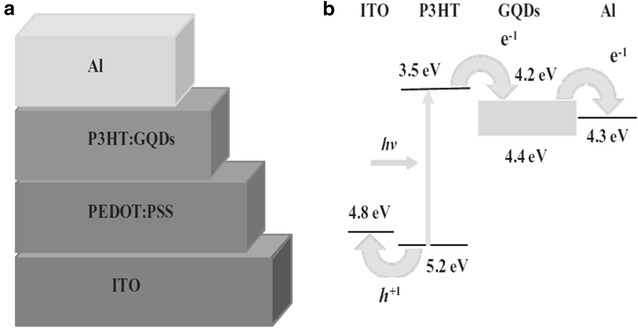
**a** Schematic and **b** energy band diagrams of an indium tin oxide (ITO)/poly(3,4ethylenedioxythiophene):poly(styrenesulfonate) (PEDOT:PSS)/poly-3-hexylthiophene (P3HT):graphene quantum dots (GQDs)/Al device. Reprinted with permission from Li et al. [63], © 2011, Wiley

**Fig. 6:**
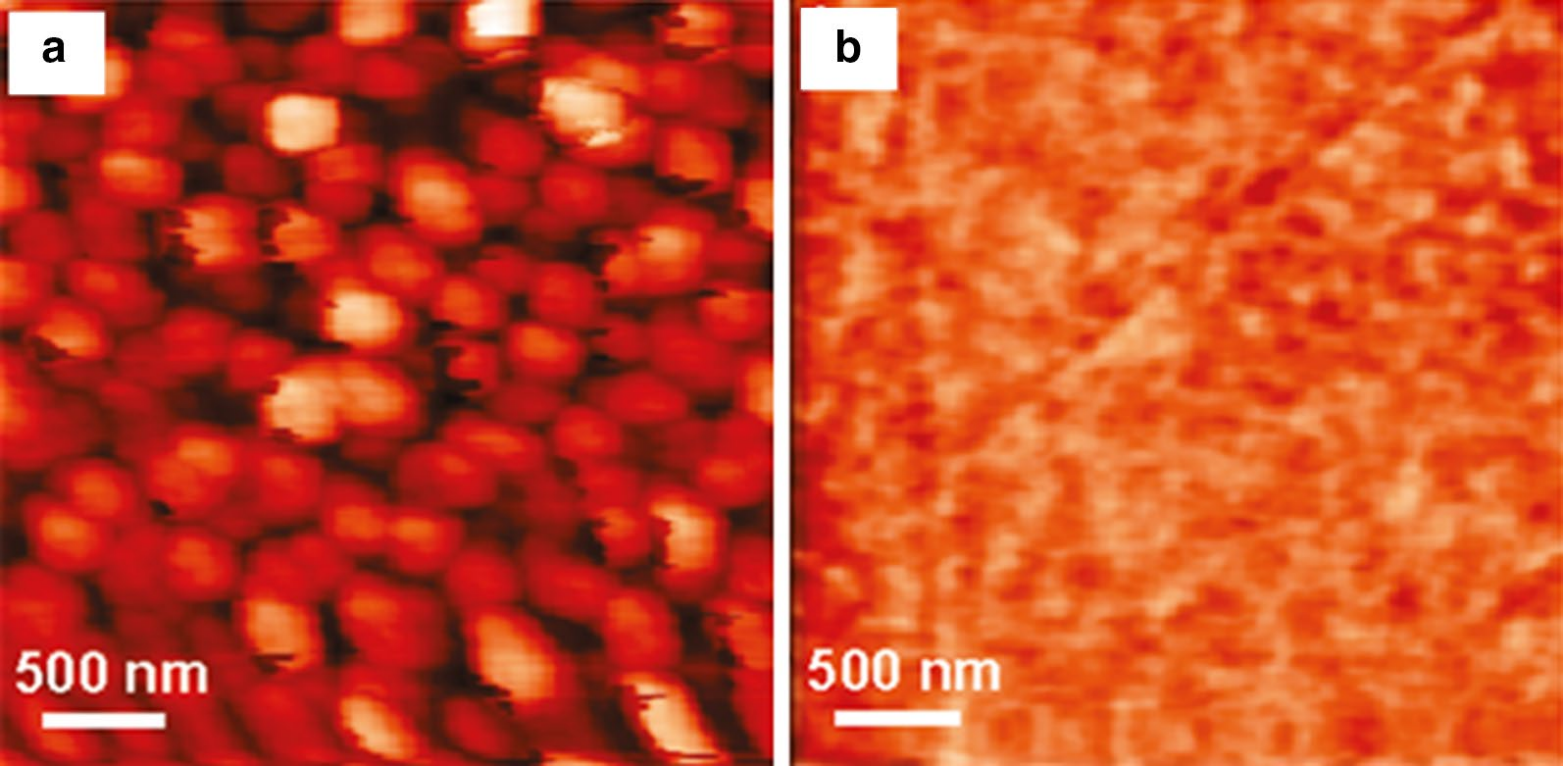
Atomic force microscopy (AFM) images of **a** graphene/poly-3-hexylthiophene (P3HT) and **b** graphene quantum dots (GQDs)/P3HT. Reprinted with permission from Gupta et al. [60], © 2011, ACS Publication

Zero-dimensional graphene quantum dots (GQDs) have also emerged as promising for solar cell applications. Due to the quantum confinement effect, GQDs show the semiconducting properties, which are different from those of graphenes [50–56]. In addition, the bandgap or energy level of GQDs can be tuned by altering the size, edge, and functional groups [57–62]. This tunability of GQD bandgaps and energy levels can potentially lead to better performance in OSCs. Several research groups have reported the advantages of using GQDs in solar cell applications. First, GQDs can enhance light absorption in solar cells. For example, Yan et al. [50] reported that GQDs exhibit a high absorption coefficient (~10^5^ M^−1^ cm^−1^) due to their bandgap. Second, GQDs facilitate the formation of charge pathways without charge recombination. Li et al. [63] reported that the incorporation of GQDs in an OSC photoactive layer enables the selective transport of electrons, as shown Fig. 5. Lastly, the smaller size of GQDs compared to graphene sheets enables more effective exciton dissociation in OSC photoactive layers. The atomic force microscopy (AFM) image of graphene/P3HT shows large features, with sizes of ~100–200 nm, but GQD/P3HT has much smaller features (Fig. 6). Thus, nanophase separation in GQDs/P3HT is closer to ideal than in graphene/P3HT, thereby leading to better PCE (~1.3 %) [60]. Recently, Hong et al. [64] reported the incorporation of small amounts of GQDs (0.01–0.08 wt%) in the PTB7:PC_71_BM BHJ blend solution and demonstrated 15 % enhancement of PCE (7.60 %) due to enhanced light absorptivity and carrier conductivity by incorporation of GQDs.

### Integration of carbon nanomaterials in OSC charge transport layer

In general, OSCs contain either a hole or electron transport layer, or both, depending on the OSC structure. In a normal OSC geometry, poly(3,4ethylenedioxythiophene):poly(styrene sulfonate) (PEDOT:PSS) is used to match the Fermi level of the transparent anode to the polymer’s highest occupied molecular orbital (HOMO) energy level and to transport holes from the photoactive layer to the transparent anode. Indium tin oxide (ITO) and Al are used as a transparent anode and metal cathode, respectively. In an inverted OSC structure, a high work function metal (e.g., Ag, Au) is used as the metal anode, ITO is the transparent cathode, and a metal oxide (e.g., ZnO, TiOx) is used as the electron transport layer and for blocking of holes from the photoactive layer to the transparent cathode [65–68]. In both normal and inverted OSCs, CNTs and graphenes can be deployed as an interlayer between the photoactive layer and the transparent electrode. The theoretical and experimentally-determined CNT work function is ~5.0 eV [69], and that of RGO is ~4.8 eV [47]. These work function values are close to that of ITO (~4.8 eV), which indicates CNTs and graphenes can transport charges from photoactive layers to transparent electrodes without an energy level mismatch.

In normally-structured OSCs, CNTs networks [70] or films [71] have been used for hole transport from the photoactive layer to the ITO anode. For example, Chaudhary et al. [70] reported about 20 % enhancement in the PCE compared to standard OSCs by placing a highly conductive CNT network on ITO and PEDOT:PSS. However, the insertion of a CNT network between a photoactive layer and the Al electrode resulted in a drastic drop in the PCE due to the energy-level mismatch between CNTs and Al metal. CNT/polymer nanocomposites as a hole transport layer was reported by Jin et al. [72]. CNT/PEDOT:PSS nanocomposites fabricated with non-covalently functionalized CNTs exhibited electrical conductivity ~2.5 times higher than that of raw PEDOT:PSS and similar optical transparency. Due to better hole transport, OSCs made using CNT/PEDOT:PSS nanocomposites exhibited PCE enhancement of ~30 % compared to OSCs made with raw PEDOT:PSS only.

**Fig. 7:**
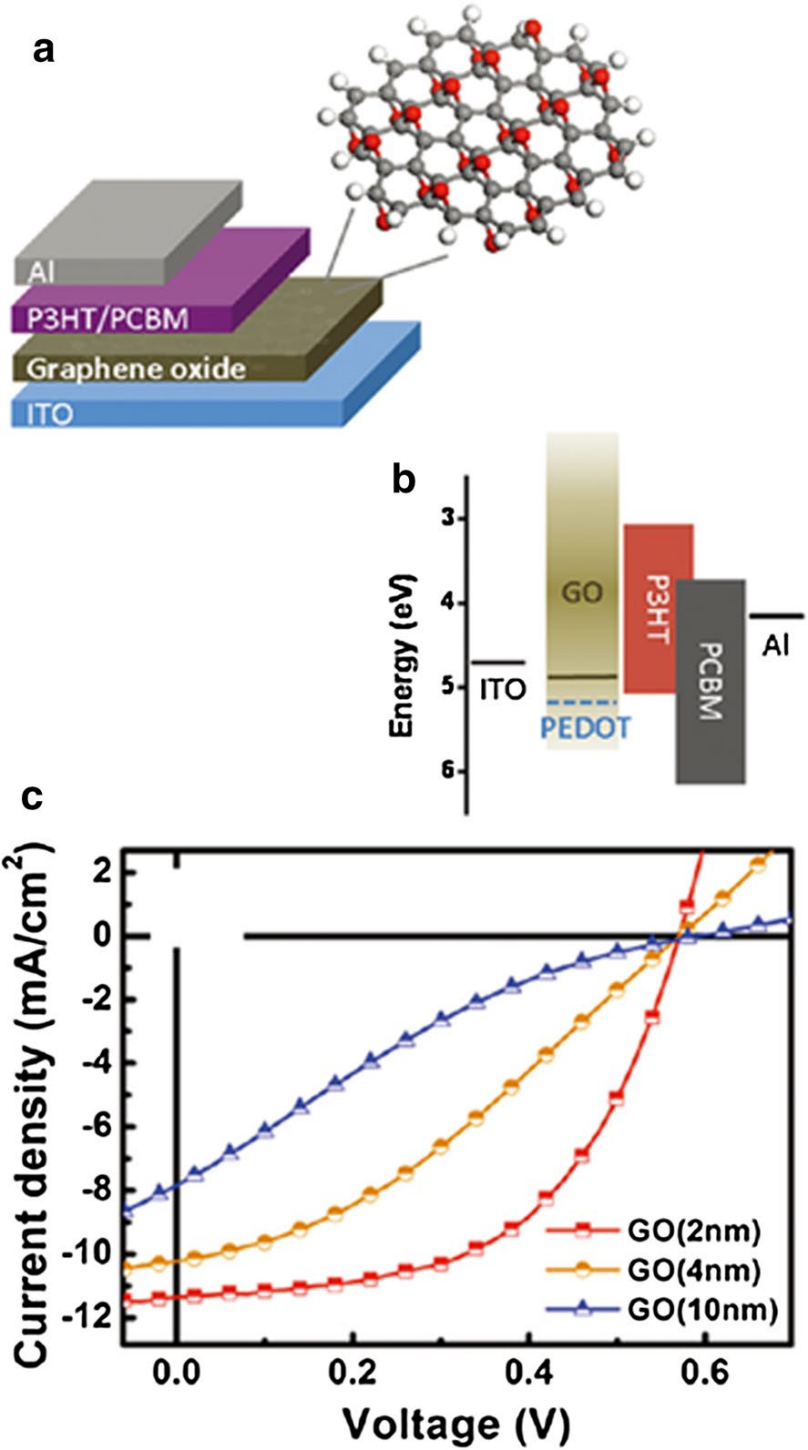
**a** A schematic diagram of an organic solar cell (OSC) with the following structure: indium tin oxide (ITO)/graphene oxide (GO)/poly-3-hexylthiophene (P3HT):6,6-phenyl-C_61_-butyric acid methyl ester (PCBM)/Al, **b** energy level diagrams, and **c** J–V characteristics of OSCs with different GO thicknesses under simulated AM 1.5 illumination at 1 sun. Reprinted with permission from Li et al. [76], © 2010, ACS Publication

**Fig. 8:**
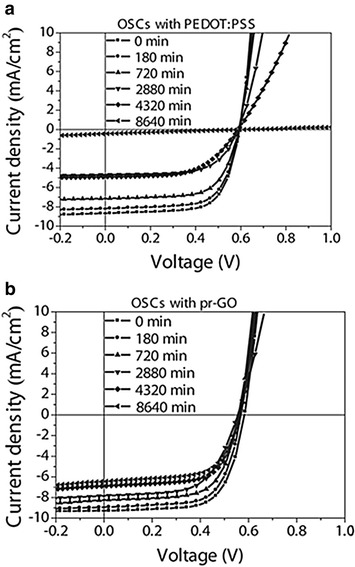
Time-dependent photovoltaic properties of **a** a conventional poly(3,4ethylenedioxythiophene):poly(styrenesulfonate) (PEDOT:PSS)-based organic solar cell (OSC) and **b** an OSC with a reduced graphene oxide (RGO) hole transport layer. Reprinted with permission from Yun et al. [77], © 2011, Wiley

Graphene also can be used as a hole transport material in OSCs. PEDOT:PSS, the conventional hole transport material, limits the long-term stability of OSCs [4, 65–67]. The high acidity of PEDOT:PSS can cause chemical instability at the interface between PEDOT:PSS and ITO [40, 73], and the inherent hygroscopic properties of PEDOT:PSS can degrade the properties of both PEDOT:PSS and the photoactive materials during ambient air exposure. Graphenes, on the other hand, are less air-sensitive and exhibit sufficient optical transparency, making them good candidates for PEDOT:PSS substitution in hole transport layers in OSCs. In addition, the simple solution processability of GO and RGO makes these materials more suitable for use in solution-processable OSCs than other inorganic hole transport materials like V_2_O_5_ and MoO_3_ [74, 75], which require the use of vacuum deposition methods. Li et al. [76] first reported the utilization of graphene as a hole transport layer in an OSC; in this work, GO with semiconducting properties (bandgap of 3.6 eV) arising from mixed sp^2^ and sp^3^ domains was used, and it can transfer holes and block electrons from the photoactive layer to the ITO (Fig. 7a, b). This device exhibited a PCE (~3.5 %) similar to that of conventional OSCs made with PEDOT:PSS hole transport layers. However, because of the insulating properties of GOs, the PCE gradually decreased with increases in the thickness of the GO film (Fig. 7c). In another report, Yun et al. [77] utilized RGO as an alternative to PEDOT: PSS for the hole transport layer in OSCs. Devices with RGO hole transport layers exhibited an average PCE of 3.63 %, which closely approaches the PCE of conventional OSCs made with PEDOT:PSS. Yun et al. also compared the lifetimes of OSCs with the conventional PEDOT: PSS and RGO hole transport layers. As shown in Fig. 8, the PCE of OSCs made using PEDOT:PSS decreased markedly after air exposure for 8640 min because of PEDOT:PSS degradation. In contrast, the PCE of OSCs with RGO hole transport layers retained 64 % of the original efficiency value after air exposure for the same duration. Graphene/polymer nanocomposites as a hole transport layer were also reported by several groups. Bae et al. reported 23 % enhancement of PCE by using poly (styrene sulfonic acid) grafted with polyaniline (PSSA-g-PANI) and GO nanocomposites compared to conventional PEDOT:PSS hole transport layer [78]. Oh et al. [79] also reported 6.59 % of PCE by using a blend of (poly[(9,9-bis((60-(N,N,N-trimethylammonium)hexyl)-2,7-fluorene)-alt-(9,9-bis(2-(2-(2-methoxyethoxy) ethoxy)ethyl)-9-fluorene))] dibromide (WPF-6-oxy-F) and graphene oxide as a hole transport layer in PTB7:PC71BM-based OSC. Since these graphene/polymer nanocomposites show higher conductivity at similar optical transparency than conventional PEDOT:PSS, they can transport the holes more effectively from the photoactive layer to the electrode without loss of optical absorption.

**Fig. 9:**
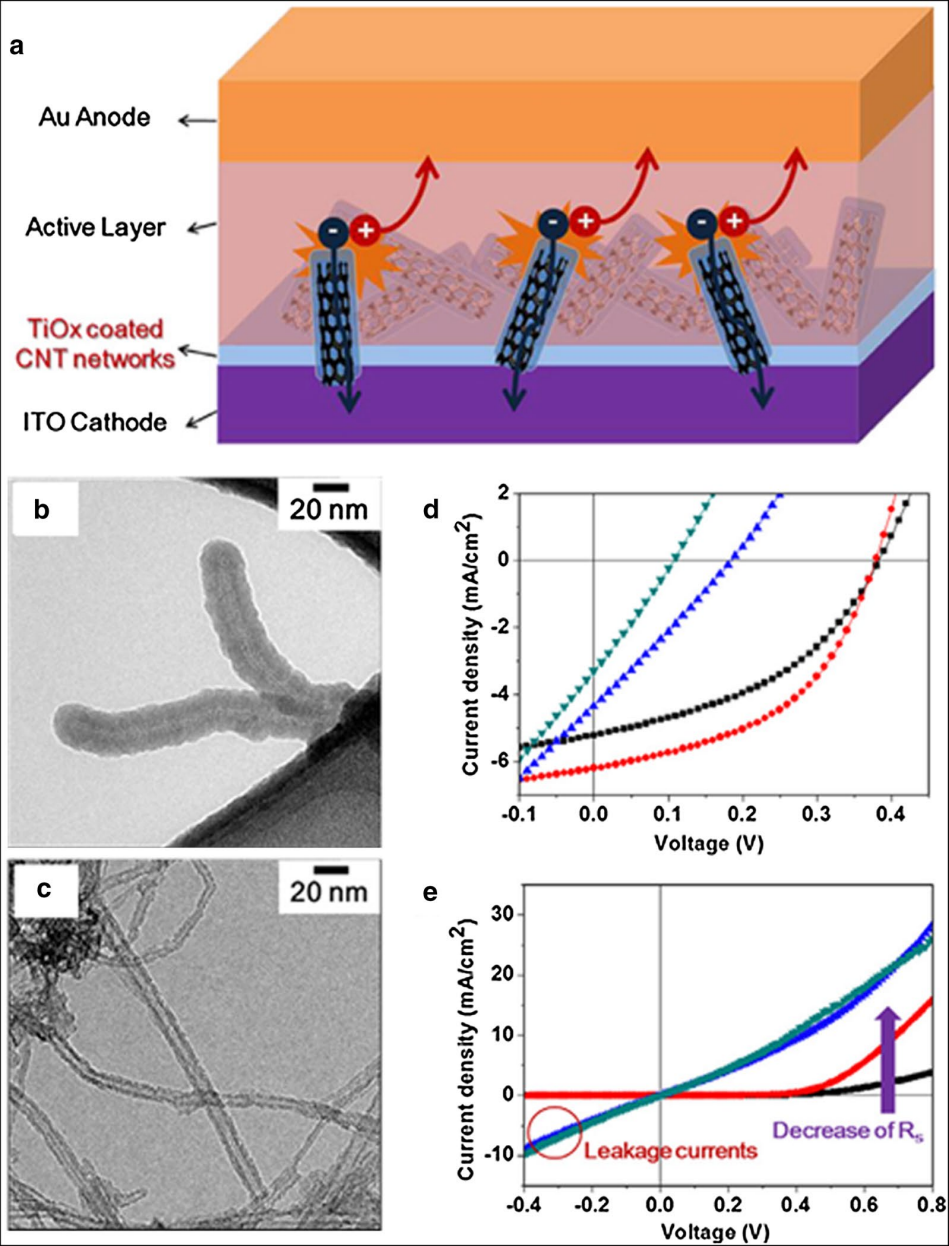
**a** A schematic diagram of TiOx-coated carbon nanotube (CNT) networks as an electron transport layer in an inverted organic solar cell (OSC), **b** transmission electron microscopy (TEM) images of functionalized carbon nanotubes (CNTs), **c** CNTs coated with TiOx using atomic layer deposition (ALD), and J–V characteristics of an inverted OSC, **d** under 1 sun illumination and **e** without illumination. Reprinted with permission from Jin et al. [80], © 2012, Elsevier

Because of the relative novelty of inverted OSC structures, fewer reports related to the use of CNTs and graphenes as electron transport layers in inverted OSCs exist than for the use of these materials as hole transport layers in normally-structured OSCs. Jin et al. [80] reported the use of TiOx-coated CNT nanocomposites as an electron transport layer in OSCs. The high conductivity of CNTs can enable good electron transport in inverted OSCs, but the metallic properties of CNTs can also cause large leakage currents, which result in a lower PCE. The coating of CNTs with TiOx, which has electron transport and hole blocking properties, enables charge selectivity. Inverted OSCs with TiOx-coated CNTs exhibited a 30 % PCE enhancement compared to inverted OSCs with TiOx only. Nanoscale conformal coating of CNTs with TiOx by atomic layer deposition (ALD) is critical for enhancing the PCE of inverted OSCs. Inhomogeneous or thin coatings of TiOx formed via sol–gel deposition causes large leakage currents, which tend to dramatically lower the PCE of inverted OSCs (Fig. 9). Hu et al. [81] also reported graphene/inorganic nanocomposites as an electron transport layer in inverted OSC. By using a ZnO/poly (N-vinylpyrrolidone) (PVP)-RGO layer as an electron transport layer, they showed enhancement of PCE from 6.8 to 7.5 % due to the higher electron transport property of ZnO/PVP-RGO than that of standard ZnO.

**Fig. 10:**
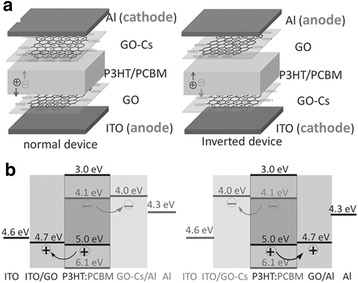
**a** Device structures and **b** energy level diagrams of normal and inverted organic solar cells (OSCs) with graphene oxide (GO) hole transport layers and GO–Cs electron transport layers. Reprinted with permission from Liu et al. [82] © 2012, Wiley

Utilization of GO for both hole and electron transport layers in the same device was reported by Dai et al. [82]. The work function of GO was shifted from 4.7 to 4.0 eV by doping with Cs (GO–Cs). GO can transport holes in normally-structured OSCs and electrons in inverted OSCs. In contrast, because of its lower work function, GO–Cs can transport electrons in normally-structured OSCs and holes in inverted OSCs, as shown Fig. 10. This result shows that energy level tuning of graphene by doping expands the possibilities for using graphene in OSC charge transport layers.

## Conclusions

In this review, recent progress toward the integration of CNTs and graphenes into OSCs was summarized. The review focused on two OSC layers: the photoactive layer and the charge transport layer. For the photoactive layer, CNTs and graphenes act as electron acceptors in combination with electron-donating polymers. The addition of CNTs and graphenes with ideal BHJ structure and energy level tuning of CNTs and graphenes are key factors for enhancing the performance of OSCs. For the charge transport layer, CNTs and graphenes have excellent potential to enhance the performance of conventional OSCs due to their high conductivity. In particular, the air-stable nature of graphenes makes them attractive for replacing PEDOT:PSS in OSC transport layers to improve long-term device stability.

The following factors require additional study for further enhancement of PCE in OSCs using carbon nanomaterials. First, the establishment of monodispersity of the energy level or bandgap of CNTs and graphenes is required. Currently, CNTs and graphenes exhibit a broad range of energy levels, and bandgaps depend on the size, chirality, doping, and functionalization, all of which lead to undesirable charge pathways and limit further PCE enhancement. The sorting of CNTs and graphenes with proper energy levels or bandgaps should, thus, be developed further. Second, wider availability of higher quality carbon nanomaterials is needed. Defects in CNTs and graphenes not only lower their conductivity but also lead to charge trapping in OSCs. Fabrication of high-quality graphene via exfoliation of graphite without oxidation and minimization of the defects in CNTs and graphenes during functionalization should be optimized. Once these challenges are met, the potentially superior properties of CNTs and graphenes compared to conventional organic materials are anticipated to enable remarkable PCE enhancement in OSCs, thereby making OSCs a realistic energy source.

## Abbreviations

CNTs: carbon nanotubes
OSC: organic solar cell
PCE: power conversion efficiency
SWNTs: single-walled CNTs
MWNTs: multi-walled CNTs
GOs: graphene oxides
RGOs: reduced GOs
PCBM: 6,6-phenyl-C_61_-butyric acid methyl ester
P3HT: poly-3-hexylthiophene
P3OT: poly-3-octylthiophene
BHJ: bulk heterojunction
TEM: transmission electron microscopy
N-CNTs: nitrogen-doped CNTs
B-CNTs: boron-doped CNTs
N-RGO: nitrogen-doped RGO
GQDs: graphene quantum dots
AFM: atomic force microscopy
PEDOT:PSS: poly(3,4ethylenedioxythiophene):poly(styrenesulfonate)
HOMO: highest occupied molecular orbital
ITO: indium tin oxide
GO-Cs: Cs doped GO
PC_71_BM: 6,6-phenyl-C_71_-butyric acid methyl ester
PTB7: poly[[4,8-bis[(2-ethylhexyl)oxy] benzo[1,2-b:4,5-b′] dithiophene-2, 6-diyl] [3-fluoro-2-[(2-ethylhexy)carbonyl]thieno[3,4-b]thiophenediyl]]
